# Multimodal imaging reveals transient liver metabolic disturbance and sinusoidal circulation obstruction after a single administration of ketamine/xylazine mixture

**DOI:** 10.1038/s41598-020-60347-1

**Published:** 2020-02-27

**Authors:** Fang-Hsin Chen, Ching-Fang Yu, Chung-Lin Yang, Yu-Chun Lin, Gigin Lin, Chun-Chieh Wang, Huang-Ping Yu, Jui Fang, Ning-Fang Chang, Ji-Hong Hong

**Affiliations:** 1 Department of Medical Imaging and Radiological Sciences, Chang Gung University, Taoyuan, Taiwan; 2 Department of Radiation Oncology, Chang Gung Memorial Hospital Linkou Branch, Taoyuan, Taiwan; 3 Radiation Biology Research Center, Institute for Radiological Research, Chang Gung University/Chang Gung Memorial Hospital Linkou Branch, Taoyuan, Taiwan; 4 Department of Medical Imaging and Intervention, Chang Gung Memorial Hospital Linkou Branch, Taoyuan, Taiwan; 5 Imaging Core Laboratory, Institute for Radiological Research, Chang Gung University/Chang Gung Memorial Hospital Linkou Branch, Taoyuan, Taiwan; 6 Clinical Metabolomics Core Laboratory, Chang Gung Memorial Hospital Linkou Branch, Taoyuan, Taiwan; 7 Department of Anesthesiology, Chang Gung Memorial Hospital Linkou Branch, Taoyuan, Taiwan; 8 Department of Biomedical Engineering, Chang Gung University, Taoyuan, Taiwan

**Keywords:** Metabolism, Preclinical research

## Abstract

A ketamine/xylazine (K/X) mixture is widely used before and during experiments in rodents. However, the impact of short-term use of K/X mixture and its influences on data interpretation have rarely been discussed. In this study, we administered one shot of a K/X mixture and examined acute hepatic responses using biochemical analysis, histopathological examination, and non-invasive imaging to determine the delay required prior to further assessment of the liver to avoid confounding effects triggered by anaesthesia. After the K/X injection, aspartate aminotransferase (AST) in serum was significantly elevated from 3 to 48 h. Obstructed sinusoidal circulation lasting for 24 or 36 h was revealed by DCE-MRI and drug distribution analysis, respectively. Metabolic alterations were detected at 3 h by NMR analysis and FDG-PET. Moreover, ultrasonography showed that lipid droplet accumulation increased from 1 to 16 h and declined thereafter. Taken together, our findings show that the K/X mixture induces acute hepatotoxicity and metabolic disturbance, and these disturbances cause hemodynamical disorders in the liver. The required time interval for recovery from K/X impact was dependent on the chosen assay. It took at least 16 h for metabolic recovery and 36 h for recovery of sinusoidal circulation. However, the liver was not fully recovered from the injury within 48 h.

## Introduction

Ketamine, a non-competitive antagonist to the *N*-methyl-D-aspartate (NMDA) receptor, is extensively applied as an adjunct anaesthetic in clinical practice and animal studies for its anaesthetic and analgesic properties, but has limited functionality in muscle relaxation^1,2^. Xylazine, an alpha-2-adrenergic central nervous system agonist, serves as a supplement to ketamine; it has analgesic and sedative effects as well as muscle relaxant properties^3^. Co-administered ketamine and xylazine has been shown to provide safe anaesthesia and sedation for small rodents in a dose-dependent manner^4^. Although the ketamine/xylazine (K/X) combination is commonly used for anaesthesia in rodents, negative side effects can occur across different species in various types of cells, such as hepatocytes, neurons, cardiac and respiratory tissues, as well as the urinary system^5–11^. These side effects are especially concerning when K/X is administered with a high dose and in cases with long-term use.

Ketamine and xylazine are both exclusively metabolized by hepatic enzymes into over 20 metabolites, which are excreted through urine and bile^12,13^. Liver injuries, particularly those induced by ketamine, have been extensively studied with a focus on hepatocytes and other remnant cells. Wai *et al*. showed that long-term use of ketamine induced liver injury through fatty degeneration and fibrosis^9^. Prolonged administration of ketamine has also been shown to cause histopathological changes, such as sinusoidal and biliary dilation as well as mitochondrial degeneration of the liver^8,14^. Elevated serum levels of alanine transaminase (ALT) and aspartate transaminase (AST) are additional evidence of hepatotoxicity induced by repeated ketamine administration^15^. However, in many animal studies, anaesthesia is only administered once or over a short-term during experiments. Further, the acute responses in rodents after short-term K/X use and how these effects influence data interpretation have rarely been discussed. Machado *et al*. reported that K/X caused serum haemolysis and marked hepatic glycogenolysis in Wistar rats after achieving profound anaesthesia at 5 min^16^. In ICR mice, administration of K/X has been shown to significantly induce hepatic injury, revealed by elevated AST activity and apoptosis of Kupffer and endothelial cells at 3 h^17^. Currently, there is a paucity of information describing the effects of K/X on hepatic parenchymal cells, and the effect of K/X on hepatic metabolism and blood flow in hepatic sinusoids has not been described. Since anaesthesia is a necessary step for experimental procedures in animals, either prior to sample collection or during medical imaging, we hypothesized that administering K/X anaesthesia may mask acute effects if liver tissue is the experimental focus. In this study, we mainly used C57BL/6J mice, a strain commonly used as an animal model, to evaluate the acute toxic effects of K/X on the liver using biochemical analysis, histopathological examination, and metabolomics profiling. C3H and BALB/c mice were also used to examine the histopathological changes. Since imaging modalities are gaining widespread use for evaluating experimental end-points, we also studied the effects K/X may introduce as background noise during ultrasonic and fluorodeoxyglucose positron emission tomography (FDG-PET) scanning and on hepatic blood flow for contrast agent delivery during dynamic contrast-enhanced magnetic resonance imaging (DCE-MRI) scans. We report that K/X acutely affected histopathology, hepatic enzymes, metabolism, and the sinusoidal blood supply of liver tissue.

## Results

### Histopathological changes in liver after anaesthesia

**Figure 1:**
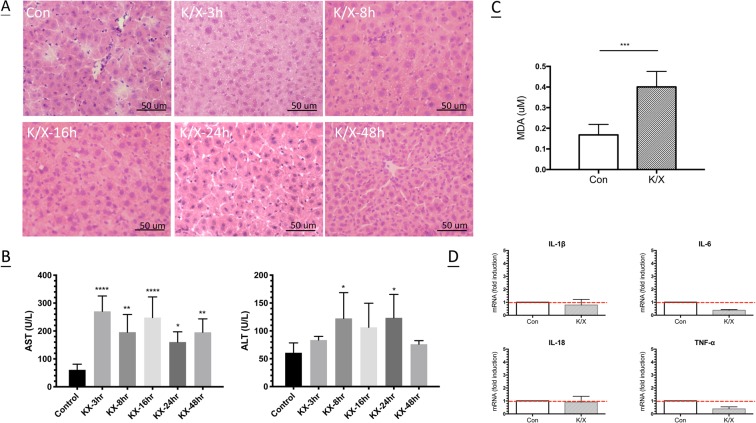
Histopathological features and liver injuries after anaesthesia. (**A**) Liver were obtained from control mice and at 3 h, 8 h, 16 h, 24 h and 48 h after mice exposure to K/X, and stained with hematoxylin and eosin to identify the liver histopathology. Representative images show significant hepatocytes swelling and congestion in hepatic sinusoidal space during 3 h to 16 h post K/X treatment (*n* = 3/group). Magnification: 400× and scale bar: 50 um. (**B**) Serum levels of ALT and AST after exposure to K/X mixture. Values are expressed as mean ± SD (*n* = 5/group, *P < 0.5, **P < 0.01, ***P < 0.001, ****P < 0.0001). (**C**) Hepatic levels of MDA at 3 h after exposure to K/Xmixture. Values are shown as mean ± SEM (*n* = 5/group, **P < 0.001, ***P < 0.0003). (**D**) mRNA level of IL-1β, IL-6, IL-18 and TNF-α in liver tissue at 3 h after exposure to K/X mixture were assessed using real-time RT-PCR analysis. Values are expressed as mean ± SEM (*n* = 5/group).

We harvested livers for histopathological examination at selected time points after mice were anaesthetised with K/X. K/X triggered significant changes in liver histology; the cellular size of hepatocytes increased and congestion in hepatic sinusoidal spaces was observed over the period of 3 h to 16 h post-K/X treatment (Fig. 1A). Liver damage was also assessed after anaesthesia by measuring serum levels of albumin, alkaline phosphatase (ALP), AST, and ALT (Fig. 1B). The levels of albumin and ALP remained stable after mice were exposed to K/X (data not shown). In contrast, the level of AST was significantly elevated at 3 h to 48 h after K/X treatment compared to control mice, and ALT changes were observed at 8 h and 24 h.

### Anaesthesia induced oxidative stress in the liver

To investigate whether K/X induces oxidative stress, we measured the level of MDA in the liver. Three hours after exposure to K/X, MDA levels were significantly increased compared with the control (*P* < 0.001) (Fig. 1C).

### Expression of pro-inflammatory cytokines in the liver after anaesthesia

To investigate whether liver injuries induced by K/X were associated with inflammatory processes, the mRNA expression levels of IL-1β, IL-18, IL-6, and TNF-α in liver samples were analysed using real-time PCR. Three hours after exposure, K/X did not stimulate hepatic levels of the four cytokines. Instead, K/X mildly suppressed the inflammatory response via down-regulation of IL-6 and TNF-α, although these changes did not reach significance (Fig. 1D).

### Effects of anaesthesia on liver metabolism

**Figure 2:**
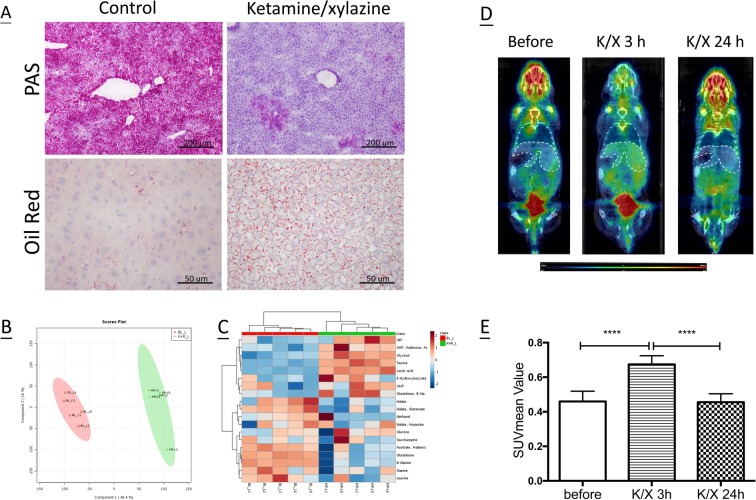
Acute metabolic disturbance induced by K/X anaesthesia. Liver samples were obtained from control and K/X mixture treated mice at 3 h after anesthesia. **(A**) Glycogen deposition was evaluated by PAS staining. Upper, magnification: 100× and scale bar: 200 um. Steatosis was assessed by ORO staining. Bottom, magnification: 400× and scale bar: 50 um. (**B**) PLS-DA of untargeted metabolomics spectra from liver tissues collected before and post K/X treatment. Two dimensional PLS-DA score plot revealed separation in metabolite profile induced by K/X anesthesia. Ellipses represented the 95% confidence interval. (**C**) Changes in metabolites levels was illustrated using heatmap. Each row represents a liver sample and each column represents the expression level of a metabolite. Data are expressed as fold change between control and K/X-treated mice and shown in a color-coded way. Red color represents an increase, and blue color a decrease. (*n* = 5/group). (**D**) CT and FDG-PET scan were performed prior to, at 3 h and 24 h post K/X treatment and showed tracer retention in the liver (outline by white dotted line), especially at time of three hour. (**E**) Quantitative analysis by SUVmean value showed a profound increase of FDG signal at 3 h, but not at 24 h post K/X treatment (*n* ≥ 5/group, ****P < 0.0001).

To further define anaesthesia-induced liver injuries, we examined glycogen storage and hepatic steatosis in liver samples. K/X treatment markedly reduced glycogen storage and in its place caused deposition of extensive lipid droplets in liver sections, leading to liver steatosis at 3 h post treatment (Fig. 2A). In order to further elucidate metabolic disturbances induced in the liver by K/X, we examined the metabolome of liver lysates at 3 h post K/X using ^1^H NMR. The K/X group was discriminated from the control group along the principle component 1 (PC1) direction (Fig. 2B). Specifically, we observed significantly increased levels of IMP, AMP, UMP, glycerol, taurine, lactic acid, 3-hydroxybutyrate, and glucose, and significantly reduced levels of malate, glutamate, glutathione, β-alanine, and leucine (using the VIP score rankings) with K/X anaesthesia compared to the control. We generated a heat map based on Euclidean distances and the Ward clustering algorithm (Fig. 2C) to illustrate the fold changes in each metabolite induced by K/X treatment relative to their usual abundance in liver tissues. After correcting for the false discovery rate, metabolites with a significant difference in concentration level (*P* < 0.05) were further mapped onto relevant pathways, including pathways involving pyruvate metabolism, glycolysis or gluconeogenesis, taurine and hypotaurine metabolism, beta-alanine metabolism, glutathione metabolism, and purine and pyrimidine metabolism (Table 1). Among the identified metabolic alterations, glucose metabolism was found to play a central role in the effects of K/X treatment. We therefore utilized the FDG isotope tracer, a glucose analogue, to monitor how K/X affects glucose uptake by the liver. We performed FDG-PET imaging on each mouse before and after K/X treatment. Tracer retention in the liver is shown in Fig. 2D, and mean standardized uptake values (SUVs) are shown in Fig. 2E. K/X triggered a general increase in glucose uptake (1.67-fold; *P* < 0.001) at 3 h post-treatment, but this increase was reduced to basal levels at 24 h.

**Table 1:** Metabolic pathway between control and KX-treated group.

Pathway Name	Total Compound	Hits	Raw p	FDR	Impact
Pyruvate metabolism	23	2	1.51E-07	1.90E-06	1
Glycolysis or Gluconeogenesis	26	1	1.52E-07	1.90E-06	0
Taurine and hypotaurine metabolism	8	1	1.31E-06	8.18E-06	0.42857
Primary bile acid biosynthesis	46	1	1.31E-06	8.18E-06	0.02976
Glycerolipid metabolism	18	1	6.41E-05	0.00032068	0.28098
Pyrimidine metabolism	41	2	0.001558	0.0044254	0.09157
beta-Alanine metabolism	17	1	0.0015931	0.0044254	0.44444
Propanoate metabolism	20	1	0.0015931	0.0044254	0
Pantothenate and CoA biosynthesis	15	1	0.0015931	0.0044254	0
Glutathione metabolism	26	1	0.0024874	0.0062186	0.36069
Purine metabolism	68	1	0.01481	0.03366	0.1163

Total is the total number of compounds in the pathway; the Hits is the actually matched number from the user uploaded data; the Raw P is the original P value calculated from the enrichment analysis; the false discovery rate (FDR) is the portion of false positives above the user-specified score threshold; the Impact is the pathway impact value calculated from pathway topology analysis.

### Liver steatosis kinetics after anaesthesia

**Figure 3:**
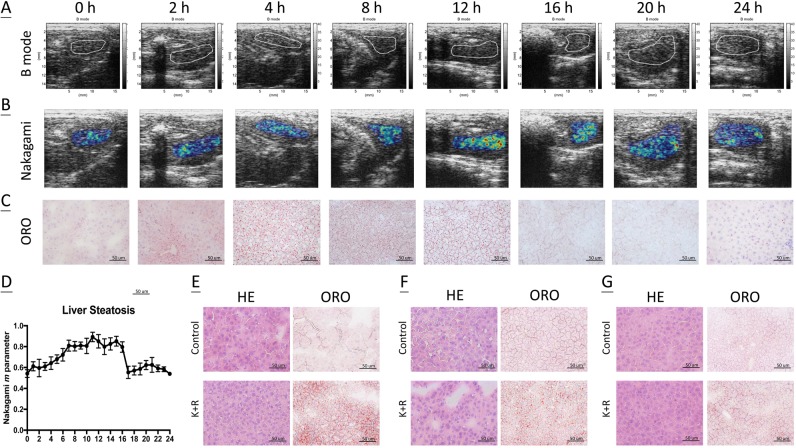
Ultrasonography visualized the liver steatosis. To find the kinetics of liver steatosis, ultrasonography was performed longitudinally on 4 mice prior to and post K/X treatment, and repeated in duplicate (*n* = 8). (**A**) Representative B mode scans. Liver was outlined by white dotted line (**B**) Representative Nakagami scans. (**C**) The liver tissues in a parallel study were harvested and evaluated by ORO staining to show the *de novo* liver steatosis (*n* = 3/group). Magnification: 400× and scale bar: 50 um. (**D**) Nakagami m parameter of the mice liver as function of steatosis score. (**E**) Representative H& E staining and liver steatosis in female C57/B6 mice at 3 h post K/X treatment (*n* = 3/group). Magnification: 400× and scale bar: 50 um. Representative H& E staining and liver steatosis in male BALB/c (*n* = 3/group) (**F**) and male C3H mice (*n* = 3/group) (**G**) at 3 h post K/X treatment. Magnification: 400× and scale bar: 50 um.

Since we identified extensive lipid droplets in liver sections at 3 h after K/X treatment, we further examined whether these changes and steatosis kinetics could be detected by ultrasonography. Livers were examined using the B mode and Nakagami imaging at one-hour intervals after K/X treatment. B mode image data from selected time points are shown in Fig. 3A; however, these images could only provide rough information on liver steatosis. Conversely, the average Nakagami parameters as quantitated by the *m* parameter indicated a gradual increase in liver steatosis from 1 h post K/X treatment, with a peak during 8 h to 16 h followed by a decline thereafter to the basal level at 24 h (Fig. 3B,D). These findings from the Nakagami images precisely matched the steatosis kinetics revealed by oil red O (ORO) stain in liver tissues in a parallel experiment (Fig. 3C), suggesting that ultrasonography is an appropriate modality to detect these changes. In addition, female C57BL/6J mice and male BALB/c and C3H mice were injected with the same dose of K/X and evaluated for morphology and extent of liver steatosis at 3 h post-K/X treatment. Similar sinusoidal congestion and accumulation of lipid droplets was also found in these mice; however, these changes had occurred to different extents, suggesting that K/X induces a general metabolic disturbance in the liver with some variation among sexes and strains of mice, as validated by ORO examination.

### Obstruction of liver sinusoidal perfusion after anaesthesia

**Figure 4:**
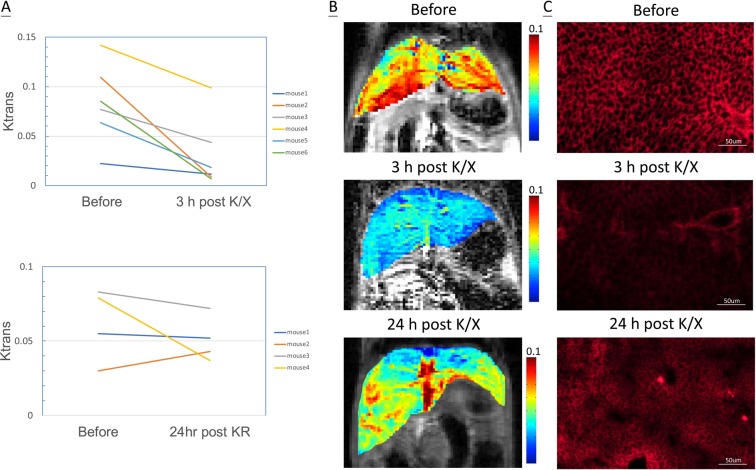
Decline of sinusoidal circulation post K/X treatment. (**A**) DCE-MRI was performed prior to and post K/X treatment at 3 h (*n* = 6). (**B**) Illustration of the K^trans^ map of a mouse (mouse#3) prior to and post K/X treatment at 3 h. (**C**) Corresponding circulation in hepatic sinusoids was identified by autofluorescence signal of Evan’s blue. Magnification: 400× and scale bar: 50 um.

We performed DCE-MRI on mice prior to K/X treatment and at 3 h and 24 h post-treatment to examine hemodynamic changes in their livers. The K^trans^ values dramatically declined at 3 h post-K/X treatment, with a mean drop to 0.37 ± 0.26-fold (Fig. 4A,B), and partially recovered at 24 h. In parallel, we evaluated sinusoidal circulation using intravenous injection of Evan’s blue to examine its influx into the liver. Figure 4C shows that Evan’s blue perfused homogeneously into liver sinusoids prior to K/X treatment; however, its signal was dramatically diminished at 3 h post-K/X treatment. The Evan’s blue perfusion recovered at 24 h but was less extensive than pre-treatment. Full recovery of dye perfusion was observed at 36 h post-K/X treatment (as shown in Supplementary Fig. 1).

## Discussion and Conclusions

The present study comprehensively documented acute hepatic injury, metabolic disturbance, and morphological and functional changes in mice after short-term use of K/X. Congestion in the hepatic sinusoidal space led to hemodynamic changes that were validated by blood perfusion analysis and DCE-MRI. Metabolic disturbances were identified by histopathological staining, ^1^H NMR, FDG-PET, and ultrasonography. Our study also revealed that short-term use of K/X caused hepatotoxicity, as confirmed by serum biochemical analysis and lipid peroxidation.

A previous *in vitro* study showed that clinically relevant or overdose concentrations of ketamine causes apoptotic insults and dysfunction in human HepG2 cells^18^. Side effects of ketamine on the liver have been reported, especially with long-term use; these effects were found to result in elevated liver enzymes or caused fatty degeneration and fibrosis^8,9^. Hepatotoxicity was also significant in patients with chronic ketamine abuse, among whom bile duct dilation and symptoms of liver fibrosis have been reported^19^. However, short-term effects of ketamine on liver have rarely been discussed. Another previous study showed dose–dependent genotoxicity of ketamine (alone or mixed with xylazine) in the blood; in contrast, genotoxicity was not seen in liver cells 24 h after a single-dose exposure, except at the maximum dose of ketamine (140 mg kg^−1^)^20^. Our IHC results demonstrate that K/X anaesthesia did not result in apoptosis of liver cells at 3 h or 24 h after exposure (data not shown). Instead, K/X suppressed the expression of TNF-α and IL-6, which is consistent with the anti-inflammatory effects of ketamine that have been reported by others^21^. The increase in AST and ALT levels observed from 3 h post-administration of K/X anaesthesia is also consistent with other studies that were conducted on ICR mice^17^. These changes may be due to lipid peroxidation, which is detected by elevated MDA level. Notably, the same dosage of K/X anaesthesia also resulted in hepatocyte enlargement and sinusoidal congestion in female C57BL/6J mice, as well as in male BALB/c and C3H mice, leading to general acute morphological changes among different sexes and strains of mice. The underlying mechanism leading to these general morphological changes may be the gradual disappearance of claudin-1, one of the adhesion molecules that forms tight junctions, during the period of 3 h to 16 h post-K/X treatment; at 24 h post-treatment, the claudin-1 was rapidly restored (as shown in Supplementary Fig. 2). Interruption of F-actin and microtubule cytoskeleton polymerization are additional mechanisms possibly mediating the effects of ketamine, and these changes have been found to occur in a time- and dose-dependent manner^22^. Our data did not support the reported absence of histopathological changes in outbred ICR mice or Wistar rats after administering a two-fold dose of ketamine (100 mg kg^−1^) mixed with a same dose (10 mg kg^−1^) or half-dose of xylazine^11,17^, suggesting that ketamine/xylazine induces model- or species-dependent liver injuries.

We identified the impact of K/X anaesthesia on liver metabolism using histopathological staining and ^1^H NMR-based metabolomic analysis. A binary switch in liver storage of glycogen and lipids was induced by K/X anaesthesia, in which glycogen storage was totally suppressed and replaced by extensive accumulation of lipid droplets, as confirmed by PAS and ORO staining. These histopathological changes were observed in liver tissue samples after single use of ketamine or xylazine at an equivalent dose; meanwhile, after administration of the K/X mixture, these changes were present to a lesser extent (data not shown). Furthermore, ^1^H NMR-based metabolomic analysis showed that K/X anaesthesia significantly altered liver metabolites compared to untreated mice. We also described abnormal metabolic profiles related to specific metabolic pathways, such as glycolysis, gluconeogenesis, TCA cycle, and energy metabolism. The high concentration of glucose we observed, accompanied by the lower concentrations of lactate related to repressed glycolysis, suggests enhanced gluconeogenesis in the K/X-treated group. Further, the important intermediate malic acid decreased as a result of K/X anaesthesia, suggesting a significant metabolic change in the TCA cycle. Leucine concentrations also changed in K/X-treated mice, suggesting that the metabolic pathway for branched-chain amino acids (BCAAs) was also altered compared to untreated mice. The present study was the first to identify biomarkers induced by short-term use of K/X anaesthesia through ^1^H NMR-based metabolome analysis.

We also evaluated the effects of K/X anaesthesia via non-invasive imaging modalities to examine changes in hepatic physiology and pathology. Together with our ORO staining results, ultrasonic scanning detected a dynamic profile of liver steatosis following administration of K/X, in which significant induction of steatosis was observed 1 h post-K/X administration, with the maximum level occurred from 8 to 16 h and a basal level of steatosis was restored thereafter. It was not a unique response in C57BL/6J mice since ORO examination revealed accumulation of lipid droplets after K/X treatment in other inbred strains of mice, such as BALB/c and C3H. PET scanning also revealed elevated uptake of glucose at 3 h post-K/X administration; this finding was consistent with the increased presence of glucose metabolites detected via NMR. In addition, DCE-MRI revealed a decline of vascular functionality in the liver from 3 to 24 h post-K/X administration; this finding was further confirmed using Evan’s dye perfusion, in which the temporary blockage in vascular functionality was partially recovered at 24 h and fully recovered at 36 h post-K/X administration. The loss of sinusoidal circulation might only be partially caused by hepatocyte swelling, since this morphological change had recovered at 16 h after K/X treatment, suggesting that additional factors are involved in the exacerbation of vascular function following K/X treatment. Notably, as it was performed with manual restraint, ultrasonography was the only scan that detected the pure effect of K/X on liver tissue. In contrast, when mice were assayed using PET or MRI to detect hepatic responses to K/X treatment, isoflurane inhalation (at least 15 min, up to 40 min) was required to maintain stable sedation. Although isoflurane alone did not trigger hepatic changes when tissue morphology, serum enzymes, MDA content, and inflammation of livers were examined (as shown in Supplementary Fig. 3), it did slightly affect liver metabolism, as shown by glycogen and lipid droplets deposition, and may intensify liver injuries when combined with the effects triggered by K/X. The effects of ketamine on hepatic blood flow have been discussed in other studies, in which blood flow in the portal vein and hepatic artery were continuously measured using inserted electromagnetic flow probes. This showed no significant difference from basal levels, even at the highest ketamine dose^23,24^. In contrast, our findings provide more detailed information regarding reduced blood flow in the sinusoidal system throughout all liver tissue. To our knowledge, our study is the first to explore the acute hepatic effects of K/X via multiple, non-invasive imaging modalities, and this revealed abnormal signals due to metabolic disturbances and obstructed vascularity.

Finally, limitations of this study include the mixed administration of ketamine and xylazine as well as the unavoidable use isoflurane inhalation during MRI and FDG-PET scanning, which may intensify obstruction of sinusoidal circulation and FDG uptake. However, we were able to clearly demonstrate that short-term use of K/X has temporary histopathological effects, such the induction of liver injuries and metabolic disturbances, and causes elevated signals of liver steatosis and glucose uptake over background when analysed using ultrasonography and FDG-PET. This suggests that the liver metabolism of any drug may be affected by previously administered K/X. DCE-MRI of the liver may also be misinterpreted under these circumstances. The obstruction of sinusoidal perfusion leads to impaired vascular functionality in the liver and may impede the delivery and distribution of drugs in pharmacological studies. Although these pathological changes are reversible in animals, further investigations with human subjects will be required to determine whether similar changes can be seen with short-term use of ketamine in humans and to determine how such changes would affect clinical assessment, especially considering the wide use of ketamine in paediatric patients in emergency departments^25^. Based on our findings, a single injection of K/X in mice triggers temporary elevation of serum enzymes, morphological, and metabolic alterations, as well as blockage of sinusoidal circulation. Since the recovery time for each physiological status is varied, we suggest that assays performed on the liver should be avoided or interpreted with caution within the first 36 h after K/X anaesthesia, and determination of serum enzymes is not suggested even 48 h after K/X anaesthesia.

## Methods

### Animals and experimental models

Male C57BL/6J mice were obtained from the National Laboratory Animal Centre, Taiwan, and used at 8–12 weeks. The study was carried out with the approval of the Institutional Animal Care and Use Committee of Chang Gung University (CGU13-080) and complied with the animal experimental guidelines laid out in the Guide for the Care and Use of Laboratory Animals. Animals received a mixture of ketamine (50 mg kg^−1^) and xylazine (10 mg kg^−1^) by intraperitoneal injection for anaesthesia.

### Serum chemistry

Serum levels of alkaline phosphatase (ALP), albumin, aspartate aminotransferase (AST), and alanine aminotransferase (ALT), were determined by an automated chemistry analyser (Siemens, ADVIA 1200) as markers of hepatic injuries.

### MDA (malondialdehyde)

The MDA in liver tissues were measured by a TABRS assay kit (Cayman) according to the manufacturer’s protocol.

### Histopathological examination

Paraffin-embedded liver sections were stained with hematoxylin and eosin (Merck) or with Periodic Acid Schiff staining (PAS, Abcam) according to standard protocol. Cryostat sections were stained with oil red O (ORO, Sigma), washed, and then counter-stained with hematoxylin (Modified Mayer’s).

### Reverse transcription polymerase chain reaction

Liver tissues were preserved to isolate RNA for RT-qPCR (Supplementary Materials).

### Vascular perfusion and MRI acquisition

The vascular perfusion in liver was examined by DCE-MRI (BrukerClinScan, 7 T MRI) prior to and after K/X anaesthesia. The pharmacokinetic analysis of Gd-DTPA (Magnevist; Bayer Schering) were performed using the Kety model^25^ for measuring the volume transfer constant (K^trans^) (Supplementary Materials). Evan’s blue (3.75 mg ml^−1^, 100ul) was intravenously injected 30 minutes before mice sacrifice for detection of vascular perfusion.

### FDG-PET and CT acquisition

Each mouse was scanned using the Inveon TM system (Siemens) prior to and after K/X anaesthesia to quantify the retention of [^18^F]fluorodeoxyglucose (FDG) in livers and followed by NanoSPECT/CT (Siemens) for anatomical registration. All image analyses were performed using the PMOD analysis software (version 3.2). (Supplementary Materials).

### Ultrasound examination and Nakagami imaging

Ultrasonic scans were performed with a commercial ultrasound scanner (SonixTouch, Ultrasonix) combined with a linear transducer (L40-8/12, Ultrasonix) operating at 11 MHz as described previously^26–31^. Briefly, mice livers were scanned prior to and every hour after K/X injection for 24 hours. During scanning, the mouse was immobilised by physical force instead of extra anaesthesia. B mode images and Nakagami images were generated as described in the Supplementary Materials.

### Quantitative analysis of liver metabolites by ^1^H-nuclear magnetic resonance (NMR)

The ^1^H-NMR spectra of liver metabolites were analyzed using a Bruker Avance 600 MHz spectrometer (Bruker) according to a published procedure^32^. The ^1^H-NMR raw data were processed by NMRProcFlow (http://www.nmrprocflow.org/), and further analysed using MetaboAnalyst 3.0 (http://www.metaboanalyst.ca) and Chenomx NMR Suite 7.5 professional software (Chenomx Inc.). (Supplementary Materials).

### Statistics

GraphPad Prism software was used for statistical analyses. For all comparisons, we assessed statistical significance by unpaired t-tests with *P value* ≦ 0.05.

## Supplementary information

Supplementary information.
