# Supplementary material for: Multimodal imaging reveals transient liver metabolic disturbance and sinusoidal circulation obstruction after a single administration of ketamine/xylazine mixture
Source: Sci Rep. 2020 Feb 27;10:3657. doi: 10.1038/s41598-020-60347-1 (PMC7046666; doi:10.1038/s41598-020-60347-1)
Supplement: Supplementary file 1 — Supplementary information. [file 41598_2020_60347_MOESM1_ESM.docx]

***Multimodal imaging reveals transient liver metabolic disturbance and sinusoidal circulation obstruction after a single administration of ketamine/xylazine mixture***

Fang-Hsin Chen^1,2,3^, Ching-Fang Yu^2^, Chung-Lin Yang^1^, Yu-Chun Lin^1,4^, Gigin Lin,^4,5,6^, Chun-Chieh Wang^1,2,3^, Huang-Ping Yu^7^, Jui Fang^8^, Ning-Fang Chang^1^, Ji-Hong Hong^1,2,3^

*Supplementary materials and methods*

*Reverse transcription polymerase chain reaction*

Total RNA was extracted from liver tissues by the Trizol reagent (Invitrogen), then reversely transcribed into cDNA with a reverse transcription kit (Qiagen) according to the manufacturer’s instructions. The cDNA was amplified using a Tag DNA Polymerase kit (Roche). Primer sequences were as follows: mouse β-actin; mouse NLRP3; mouse Casp-I; mouse IL-1β; mouse IL-18; mouse IL-6; and mouse TNF-α. The PCR program ran on a Bio-Rad system as follows: 95°C for 5 min; 95°C for 10 s; 60°C for 15 s; 72°C for 30 s (40 cycles); and 95°C for 10 s. The mRNA expression level of each target gene was quantitated by normalisation to that of β-actin.

*MRI acquisition*

MRI was performed using a 7-Tesla animal MR scanner (ClinScan, Bruker). Mice were anaesthetised during the MRI experiment with 1%–2% isoflurane in air. Body temperature was maintained at 37°C with a water-bed heating system (Model 1025, SA instrument, New York, USA). The respiration rate was kept at 30–40 cycles min^-1^. DCE MR imaging was acquired using a spoiled 3D gradient echo sequence with the following parameters: repetition time = 4.9 ms, echo time = 1.97 ms, and temporal resolution = 2.5 s. The baseline T1 values were calculated from images acquired with multiple flip angles (5°, 10°, 15°, and 20°). We acquired a series of 120 dynamic scans with a flip angle of 20°, followed by a bolus of 0.05 ml of Gd-DTPA (Magnevist; Bayer Schering) delivered using a dedicated syringe pump (PHD 2000, Harvard Apparatus, Massachusetts, USA) with a constant infusion rate of 0.5 ml min^-1^. We performed pharmacokinetic analysis using the Kety model [18]. The quantitative parameter K^trans^, which represents the volume transfer constant from the intravascular system to the extravascular extracellular space, was calculated for each voxel using a nonlinear least-squares estimation algorithm.

*FDG-PET and CT acquisition*

Mice were fasted overnight and injected with FDG (0.15 Ci kg^-1^) via the tail vein. One hour later, mice were imaged for 10 min to obtain static scanning using the Inveon TM system (Siemens), followed by NanoSPECT/CT (Siemens) for anatomical registration. The CT contrast agent (0.1 ml per mouse, ExiTron nano 6000, Miteny Biotec) was used to identify liver tissue. Mice were anaesthetised during the scanning with 1%–2% isoflurane in air. The matrix size of the microPET reconstructed image was 128 × 128 × 159 with voxels of 0.39 × 0.39 × 0.8 mm^3^ using a 2D ordered-subset expectation maximum iterative method. We applied scatter correction and random correction to all images. The volumes-of-interest (VOIs) (3 × 3 × 2.4 mm^3^) were determined in the right lobe of the liver from a coronal view using PMOD version 3.2 image analysis software. The radioactivity concentration within the VOIs was converted to standard uptake values (SUV) by multiplying body weight with injection dose. We performed FDG-PET on mice before and 3 hr after intraperitoneal injection of the ketamine and xylazine mixture.

*Ultrasound examination*

Following ketamine/xylazine injection, livers were scanned every hour for 24 hours with a commercial ultrasound scanner (SonixTouch, Ultrasonix) combined with a linear transducer (L40-8/12, Ultrasonix) operating at 11 MHz. The pulse length of the transducer was approximately 0.375 mm and the sampling rate of the radiofrequency (RF) signals was 40 MHz. Before scanning, the mouse abdomen was shaved to produce an acoustic window. During scanning, the mouse was immobilised by physical force instead of extra anaesthesia. The transducer was passed through the epigastric region and tilted at different angles against the vertical plane to seek the largest area of liver parenchyma. The focal zone was adjusted to focus on the central part of the liver and reduce the effect of beam diffraction. We performed five independent liver scans on each mouse.

For each RF image, each scan line was demodulated to build the envelope image. We generated the B-mode image using a logarithm-compressed envelope image (dynamic range = 40 dB) and constructed the Nakagami image using a non-compressed envelope image for backscattered statistics analysis. Ultrasonic Nakagami imaging based on the Nakagami parameter is a well-established technology for assessing fat in liver tissuee. Detailed principles and descriptions for calculating the parameter and carrying out imaging are provided in Supplement A. Nakagami images were superimposed onto the corresponding B-mode images to provide structural and parametric information. To avoid interference from blood vessels while calculating the mean Nakagami parameter of the liver parenchyma, we manually selected a region of interest (ROI) in the B-mode image to calculate the mean Nakagami parameter and determine the effect of anaesthesia on backscattered statistics.

The Nakagami image based on Nakagami parameter was constructed using the sliding window technology. The Nakagami parameter *m* is a shape parameter of the Nakagami statistical model and can be estimated as

$m=\frac{[E(R^{2})]^{2}}{E[R^{2}-E(R^{2})]^{2}}$ (1)

where *R* is the envelope of backscattered ultrasonic signals. In general, the backscattered statistics of the envelopes received from biological tissues can be divided into three distribution types: (i) the Rayleigh distribution caused by a high number of randomly distributed scatterers in the resolution cell of the transducer; (ii) the pre-Rayleigh distribution (with a phase lead compared to Rayleigh statistics) caused by a low scatterer concentration or the presence of scatterers in the resolution cell with randomly varying scattering cross sections with a high degree of variance; and (iii) the post-Rayleigh distribution (with a phase lag compared with Rayleigh statistics) caused by a resolution cell containing periodically located scatterers in addition to randomly distributed scatterers. A study showed that the variation of the Nakagami parameter from 0 to 1 corresponds to a change in the envelope statistics from pre-Rayleigh to Rayleigh distributions; Nakagami parameters higher than 1 indicate that the statistics of the backscattered signal conform to post-Rayleigh distributions. Consequently, the Nakagami distribution is a general model for ultrasonic backscattering.

The details of the algorithm for ultrasound Nakagami imaging can be found in previous studies. In brief, the Nakagami parameter is computed through two main steps: (i) a square window in the envelope image is used to collect local backscattered envelopes for estimating the local Nakagami parameter, which is assigned as the new pixel located in the center of the window; (ii) the window is allowed to move throughout the envelope image in one-pixel steps. Step 1 is repeated until the map of the local Nakagami parameter is constructed. Previous studies have suggested that a side length of the square window that is three times the pulse length of the transducer can simultaneously satisfy the image resolution for stable parameter estimation. In the present study, a square window with a side length of 2.1 mm (three times the pulse length of the linear array transducer) was used to construct the Nakagami image.

*Quantitative analysis of liver metabolites by ^1^H-nuclear magnetic resonance (NMR)*

The livers were perfused with 20mL PBS/EDTA buffer to remove entrained blood before harvesting and subsequently extracted by the Folch method. In brief, samples were homogenized in 1mL of methanol in H2O (1:1 ratio) and mixed with 2.5ml of chloroform and 1.25ml of H_2_O for 1min, then centrifuged at 3,000 rpm for 25min at 4°C. After centrifugation, the upper and lower layers were transferred into separate glass vials, then concentrated by speed vacuum. Samples from upper and lower layers were re-suspended in 650 μl of deuterium water containing 0.005% 3-(tri-methylsilyl)-propionic-2,2,3,3-d4 acid sodium salt (TSP) sample, and deuterium chloroform with 0.025% tetramethylsilane (TMS), respectively. Each sample was vortexed and subsequently centrifuged at 12000 × g for 5min at 4°C. The supernatant was transferred to a standard 5mm NMR tube for analysis. ^1^H-NMR spectra were acquired on a Bruker Avance 600 MHz spectrometer (Bruker) at the Chang Gung Healthy Aging Research Center, Taiwan, according to a previous published procedure [24]. The ^1^H-NMR raw data were pre-processed using NMRspec and NMRviewer, two separate applications of NMRProcFlow (http://www.nmrprocflow.org/), for the spectral bucket, spectral region exclusion, and spectral normalization [25]. The normalized graphics and univariate analyses were obtained using MetaboAnalyst 3.0 (<http://www.metaboanalyst.ca)> using Partial Least-Squares Discriminant Analysis (PLS-DA). Model fit was determined by R2 and Q2 values after performing 10-fold internal cross-validation. The most differential peaks between control and KX-treated groups were selected and further processed using Chenomx NMR Suite 7.5 professional software (Chenomx Inc., Edmonton AB, Canada) to identify metabolites and quantify them by peak area.

*Supplement Fig. 1*

*
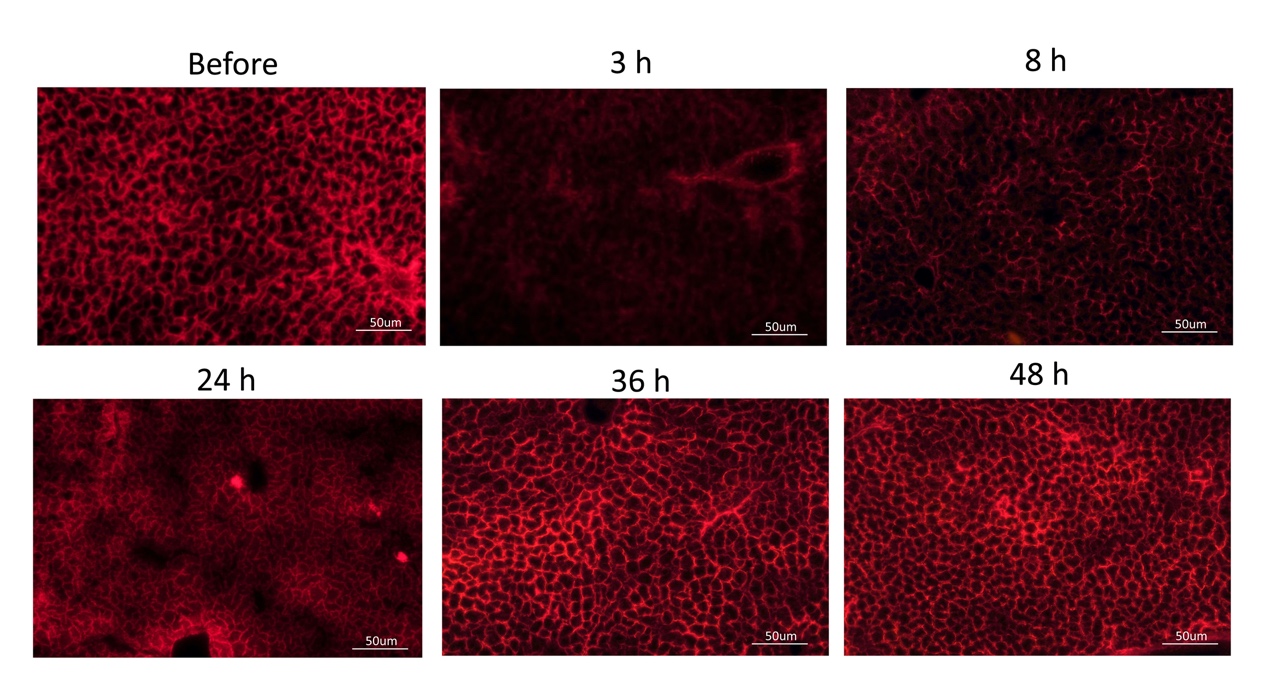
*

Dynamic sinusoidal circulation post-K/X treatment. Vascular perfusion in hepatic sinusoids was identified by autofluorescence signal of Evan’s blue at indicated times post-KX exposure. Magnification: 400× and scale bar: 50 um.

*
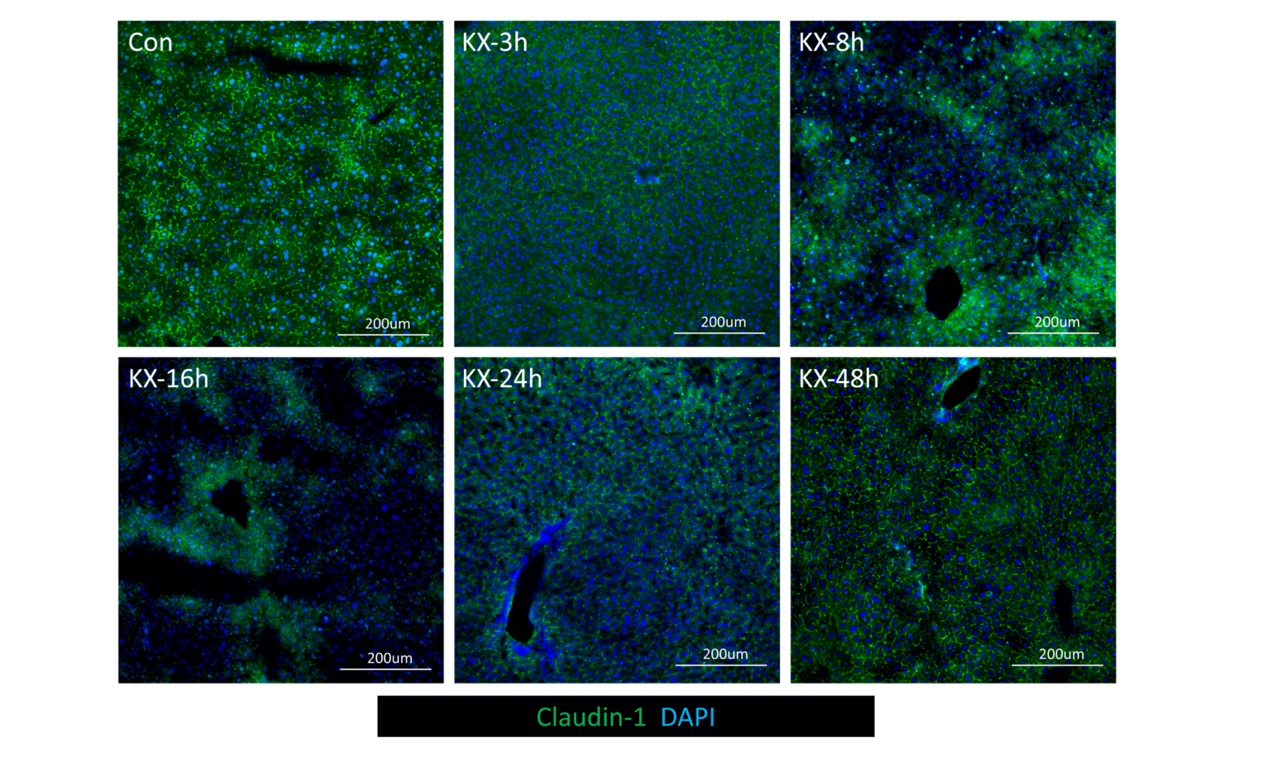
Supplement Fig.2*

Dynamic tight junction expression on hepatocytes. Representative IHC staining of liver tissues for claudin-1 at indicated times post-K/X treatment (*n*=3/group). Magnification: 400× and scale bar: 200 um. (green: claudin-1, blue: nucleus)

*Supplement Fig. 3*


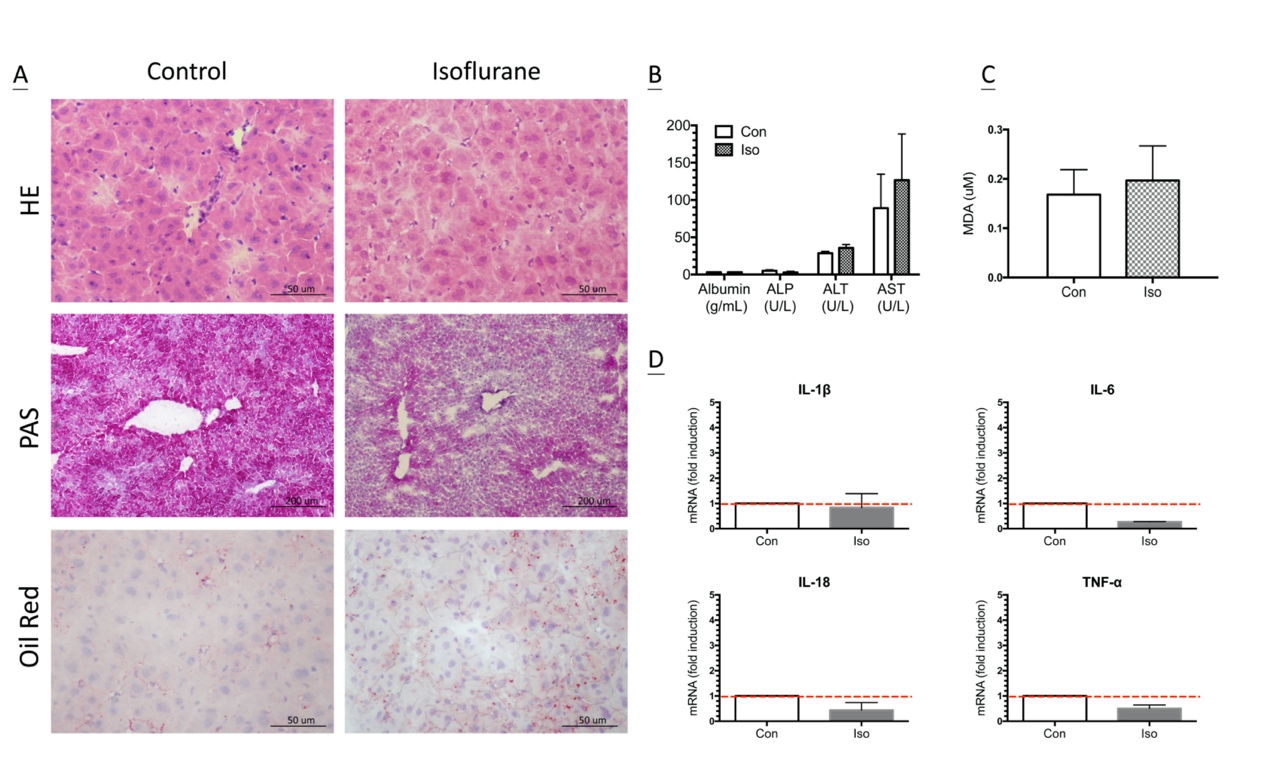


Histopathological features and liver injuries at 3 h after 20 min inhalation of 2% isoflurane. (A) Liver histopathology after exposure to isoflurane. Sections of liver were with hematoxylin (Magnification: 400× and scale bar: 50 um), PAS (Magnification: 200× and scale bar: 200 um) and ORO (Magnification: 400× and scale bar: 50 um). Representative images show similar tissue morphology in livers between non-treated and isoflurane-inhalation mice, but slight decrease of glycogen and increase of lipid droplets (*n*=3/group). (B) Serum levels of albumin, ALP, ALT and AST after exposure isoflurane. Values are expressed as mean ± SD (*n*=5/group, *********P*< 0.0001). (C) Hepatic levels of MDA after exposure to isoflurane (*n*=5/group). (D) mRNA level of IL-1β, IL-6, IL-18 and TNF-α in liver tissue were assessed using real-time RT-PCR analysis. Values are expressed as mean ± SEM (*n*=3/group).
